# Advancing Cancer Biology: Highlights from the 2025 FASEB SRC on Cellular Plasticity in Cancer

**DOI:** 10.47248/chp2502040018

**Published:** 2025-10-17

**Authors:** Hans Clevers, Ankur Sharma, Sendurai A. Mani, Anna Golebiewska, Axel Behrens, Wai Leong Tam, Loic P. Deleyrolle, Huiping Liu, Karuna Ganesh, Walid T. Khaled, Shaheen Sikandar, Terence K. Lee, Tyler E. Miller, Dean G. Tang, Sheila Singh, Vivian S.W. Li, Justin D. Lathia, Stephanie Ma

**Affiliations:** 1. Pharma, Research and Early Development (pRED) of F. Hoffmann-La Roche Ltd, Basel, Switzerland; 2. Translational Genomics Program, Garvan Institute of Medical Research, Darlinghurst, NSW, Australia; 3. Department of Pathology and Laboratory Medicine, The Warren Alpert Medical School, Brown University, Providence, RI 02912, USA; 4. Legorreta Cancer Center, The Warren Alpert Medical School, Brown University, Providence, RI 02912, USA; 5. NORLUX Neuro-Oncology Laboratory, Department of Cancer Research, Luxembourg Institute of Health, L-1210 Luxembourg, Luxembourg; 6. Cancer Stem Cell Laboratory, The Breast Cancer Now Toby Robins Research Centre, The Institute of Cancer Research, London, UK; 7. Department of Surgery and Cancer, Imperial College London, London, UK; 8. Convergence Science Centre, Imperial College London, London, UK; 9. Genome Institute of Singapore (GIS), Agency for Science, Technology and Research (A*STAR), 60 Biopolis Street, Genome, Singapore, 138672, Singapore; 10. Cancer Science Institute of Singapore, National University of Singapore, 14 Medical Drive, Singapore, 117599, Singapore; 11. Department of Molecular Medicine, Mayo Clinic, Jacksonville, FL, 32224, USA; 12. Circulating Tumor Cell Core, Robert H. Lurie Comprehensive Cancer Center, Northwestern University Feinberg School of Medicine, Chicago, IL, 60611, USA; 13. Molecular Pharmacology Program, Memorial Sloan Kettering Cancer Center, New York, NY, USA; 14. Department of Medicine, Memorial Sloan Kettering Cancer Center, New York, NY, USA; 15. Department of Pharmacology, University of Cambridge, Cambridge, UK; 16. Cambridge Stem Cell Institute, University of Cambridge, Cambridge, UK; 17. Department of Molecular, Cell and Developmental Biology, University of California - Santa Cruz, Santa Cruz, CA, 95064, USA; 18. Department of Applied Biology and Chemical Technology, Hong Kong Polytechnic University, Hong Kong SAR, China; 19. Department of Pathology, Case Western Reserve University School of Medicine and Case Comprehensive Cancer Center, Cleveland, OH, 44106-7285, USA; 20. Department of Pharmacology & Therapeutics, Roswell Park Comprehensive Cancer Center, Buffalo, NY, 14263, USA; 21. Department of Surgery, McMaster University, Hamilton, ON L8S 4L8, Canada; 22. Centre for Discovery in Cancer Research, McMaster University, Hamilton, ON L8S 4L8, Canada; 23. Department of Biochemistry and Biomedical Sciences, McMaster University, Hamilton, ON L8S 4L8, Canada; 24. Stem Cell and Cancer Biology Laboratory, The Francis Crick Institute, London, UK; 25. Department of Cardiovascular & Metabolic Sciences, Cleveland Clinic Research, Cleveland Clinic, Cleveland, OH 44195, USA; 26. Department of Molecular Medicine, Cleveland Clinic Lerner College of Medicine at Case Western Reserve University, Cleveland, OH 44106, USA; 27. Case Comprehensive Cancer Center, Cleveland, OH 44106, USA; 28. Rose Ella Burkhardt Brain Tumor and Neuro-Oncology Center, Cleveland Clinic, Cleveland, OH 44195, USA; 29. School of Biomedical Sciences, LKS Faculty of Medicine, The University of Hong Kong, Hong Kong SAR, China; 30. State Key Laboratory of Liver Research, The University of Hong Kong, Hong Kong SAR, China

**Keywords:** cancer cell plasticity, cellular plasticity, cancer stem cells, phenotypic switching, therapy resistance, tumor microenvironment, immune evasion

## Abstract

The inaugural FASEB Science Research Conference (SRC) on Cellular Plasticity in Cancer was held in May 2025 in Hong Kong SAR, China. This event brought together leading experts to discuss cutting-edge research centered on cancer cell plasticity. The conference featured comprehensive presentations covering a broad spectrum of topics, including oncofetal reprogramming in tumor development and progression, mechanisms regulating cancer cell plasticity, metabolic reprogramming and its role in tumor progression, cancer cell plasticity during metastasis, cancer stem cell programs within the tumor microenvironment, tumor plasticity and immune evasion, as well as innovative therapeutic strategies aimed at targeting stem cell-like states, modulating cancer cell states, and effectively controlling disease progression. It is anticipated that the insights gained from this meeting will catalyze further advancements in cancer biology and therapy.

## 1. Introduction and Keynote Address

Hong Kong hosted its inaugural Science Research Conference (SRC) from May 4th to 8th, 2025, at the University of Hong Kong. Centered on the theme of *Cellular Plasticity in Cancer*, this event marked a significant milestone as the first of its kind organized by FASEB. The timing of this event is particularly relevant, coinciding with the recognition of “unlocking phenotypic plasticity” as an emerging hallmark of cancer, as highlighted by Douglas Hanahan in the 2022 edition of Cancer Hallmarks [1].

Recent advances have emphasized the critical importance of targeting the stem cell states characterized by a loss of differentiation and enhanced self-renewal capabilities - an essential component for achieving long-term tumor control. Over the past decade, significant progress has been made in identifying and understanding these cellular states, driven by advancements in single-cell and spatial transcriptomics technologies. These innovations have enhanced our knowledge of cancer stemness, particularly regarding their interactions within the tumor microenvironment. The convergence of these insights suggests that developing novel therapeutic strategies with potential for clinical translation is now increasingly feasible.

***Organoids to model human disease – Hans Clevers*** from F. Hoffmann – La Roche Ltd., Switzerland, presented on the use of organoids to model human disease. His seminal work on the discovery of LGR5 as a marker for intestinal epithelial stem cells [2], along with subsequent developments in 3D organoid protocols [3] represented major breakthroughs in stem cell research, enabling the establishment of long-term, stable stem cell cultures *in vitro*. These organoids faithfully recapitulate key aspects of the organ from which they are derived. LGR5 was later identified by Clevers’ group as an exquisitely specific, yet broadly applicable, marker for active epithelial stem cells across various tissues, including hair follicles, kidneys, liver, mammary glands, inner ear, tongue and stomach epithelium. Purified single LGR5-positive stem cells can initiate and sustain the growth of organoids representing human intestine, stomach, liver, pancreas, prostate, kidney, breast and other organs *in vitro*. This development opens new avenues for studying development, physiology, disease, drug development, and personalized medicine.

Beyond LGR5-positive stem cells, many immediate progenitor cells also exhibit remarkable cellular plasticity, especially following injury. For instance, the recently discovered AVIL-positive proliferating tuft cells can survive irradiation damage and retain the ability to regenerate all epithelial cell types [4]. More recently, Clevers’ team has advanced organoid culture techniques from 3D to 2D using integrin-activating Yersinia protein, Invasin, which enables long-term expansion of epithelial cells in a 2D format [5]. This approach offers key advantages over the traditional 3D cultures, including improved imaging, functional assays, and high-throughput screening, with broad implications for biotechnology and pharmaceutical applications.

## 2. Oncofetal Reprogramming in Tumor Development and Progression

***Understanding embryonic origins of tumors – Ankur Sharma*** from Garvan Institute of Medical Research, Australia, shared his work on oncofetal reprogramming in cancers. Malignant cells are known to exhibit embryonic features; however, it was previously unclear whether cells within the tumor microenvironment, such as endothelial cells, fibroblasts, and macrophages, also display embryonic reprogramming. Using comparative single-cell transcriptomics, Sharma *et al*. [6] identified PLVAP-positive endothelial and FOLR2/HES1-positive macrophages shared between fetal liver tissue and hepatocellular carcinoma (HCC). He introduced the concept of the ‘oncofetal ecosystem’ to describe this embryonic reprogramming within the tumor microenvironment [7]. Building on this, Sharma’s team employed spatial omics techniques to characterize the oncofetal niche, which comprises POSTN-positive fibroblasts, PLVAP-positive endothelial cells, and FOLR2/HES1-positive macrophages in patient tumors. They discovered a correlation between the presence of this oncofetal niche and response to therapy in HCC [8]. Sharma also provided updates on the ongoing Phase IIb clinical trial, DEFINERx050, which aims to evaluate oncofetal cells as biomarkers for immunotherapy response. Extending these findings, he demonstrated how oncogenic mutations acquired during tumor evolution further promote oncofetal reprogramming within the tumor microenvironment, contributing to T-cell exhaustion. Additionally, he discussed the computational method SCOPE, designed to identify oncofetal cells within spatial transcriptomics data for patient stratification.

***SOX2 drives fetal reprogramming and reversible dormancy in colorectal cancer – Vivian S.W. Li*** from the Francis Crick Institute, United Kingdom, presented her recent research on cellular plasticity in colorectal cancer (CRC). Wnt signaling is hyperactivated in the majority of CRC cases, and her team employs advanced organoid technologies alongside transgenic mouse tumor models to investigate various Wnt-targeting strategies. These strategies include cell-autonomous approaches that directly target Wnt activation within cancer cells, non-cell autonomous approaches that focus on disrupting Wnt signaling mediated by the tumor microenvironment, and studies into tumor plasticity in Wnt-high CRC. Her lab previously identified USP7, a deubiquitinating enzyme, as a novel tumor-specific Wnt target in APC-mutated CRC, affecting over 80% of cases [9–10]. In recent work, Vivian Li’s team uncovered a rare SOX2-positive cell population in APC-mutated CRC that drives cellular plasticity, promoting tumor progression and drug tolerance [11].

## 3. Mechanisms Regulating Cancer Cell Plasticity

***Epithelial-mesenchymal transition (EMT)-induced stem cell properties and cellular plasticity in cancer – Sendurai Mani*** from Brown University, United States, shared his research elucidating the role of cellular plasticity in the development of metastasis, treatment resistance, and tumor relapse. These processes remain among the leading causes of cancer-related mortality, and a key mechanism driving them is the reactivation of an embryonic developmental program known as EMT. EMT enables epithelial cancer cells to acquire mesenchymal properties, enhancing their migratory and invasive capabilities, while also promoting the acquisition of cancer stem cell (CSC)-like traits. These stemness-related properties confer tumor plasticity, allowing malignant cells to self-renew, adapt to therapies, and regenerate diverse tumor populations. His team and others established a direct link between EMT and CSC states, demonstrating that inducing EMT in immortalized human mammary epithelial cells generated a subpopulation with mesenchymal characteristics and increased tumor-initiating ability [12]. This foundational study provided the first mechanistic evidence connecting EMT with the development of stemness traits, offering a model for understanding tumor plasticity across various cancer types. Recent advances in lineage tracing and single-cell transcriptomics have revealed that EMT is not simply a binary switch but exists along a spectrum, resulting in various cellular states from fully epithelial to fully mesenchymal, including hybrid epithelial/mesenchymal phenotypes. These hybrid states maintain both adhesion and migratory abilities, supporting collective invasion and increasing metastatic seeding potential. Mani and colleagues, along with others, have demonstrated that such hybrid populations may represent the most aggressive and therapy-resistant subclones within tumors, as they combine cellular plasticity with adaptability [13]. Importantly, these findings suggest that therapeutic strategies focusing only on fully mesenchymal cells may be inadequate, since hybrid EMT states serve as reservoirs of tumor-propagating cells with high metastatic capacity. At the molecular level, EMT is regulated by several transcription factors, including SNAIL, TWIST, and ZEB1/2, which suppress epithelial programs while activating mesenchymal and stemness properties. Mani’s group has emphasized the importance of these factors in maintaining CSC phenotypes and has demonstrated that EMT programs are closely linked with epigenetic and metabolic changes [14–15]. Moreover, EMT-induced plasticity is strengthened by dynamic interactions with the tumor microenvironment, including TGF-β signaling, hypoxia, and inflammatory cytokines [16]. The spatial and temporal regulation of EMT within tumors contributes to intratumoral heterogeneity, enabling adaptive responses to therapy and facilitating relapse.

Targeting EMT-mediated plasticity remains a significant challenge in oncology. Current strategies include inhibiting EMT-inducing signals, such as TGF-β pathway inhibitors, as well as Hedgehog and Wnt antagonists. While directly targeting EMT transcription factors is difficult due to their “undruggable” nature, alternative strategies involve disrupting their protein-protein interactions or targeting epigenetic regulators of EMT programs. Exploiting the vulnerabilities of EMT/CSC states, such as their metabolic dependencies, reliance on oxidative phosphorylation or lipid metabolism, also offers promising avenues. Mani’s lab has identified metabolic plasticity as a key feature of EMT-driven CSCs [17], which opens up new possibilities for therapy. Preclinical studies suggest that combining EMT-targeting strategies with existing chemotherapies, immune checkpoint inhibitors, or tyrosine kinase inhibitors may synergistically reduce tumor growth and recurrence. For example, disrupting EMT-related signaling can sensitize tumors to primary treatments and help prevent metastatic spread.

***Tumor plasticity and microenvironment shaping heterogeneity and resistance to treatment in aggressive brain tumors – Anna Golebiewska*** from Luxembourg Institute of Health, Luxembourg, presented on the role of tumor cell plasticity in creating a dynamic ecosystem within aggressive brain tumors, in particularly glioblastoma. Glioblastomas are known for their histopathological and molecular heterogeneity, comprising diverse tumor clones, phenotypic states, and a brain-specific tumor microenvironment. Numerous studies, including those from Golebiewska’s team, have demonstrated that glioblastoma cells exhibit high plasticity in response to external cues from their microenvironment [18]. Historically, the CSC field has faced challenges due to the lack of robust markers, with the identification of stem-like states often biased by shared expression within components of the tumor microenvironment [19–20] or by genetic clones [21]. Golebiewska also emphasized the strong inter-patient variability, which complicates the development of universal markers for tumor stemness. Importantly, intratumoral phenotypic heterogeneity can arise from intrinsic plastic properties of tumor cells, which operate in a stochastic, non-hierarchical manner, as evidenced by the mathematical Markov modelling [22]. Glioblastomas thus create a dynamic ecosystem where heterogeneous tumor cells interact with the microenvironment to establish various niches. The equilibrium of phenotypic states within the tumor depends on intrinsic genetic and epigenetic factors, as well as extrinsic pressures from the microenvironment. Interestingly, while all tumor cells demonstrate plasticity, some can more rapidly regain their equilibrium states than others.

Golebiewska highlighted that phenotypic plasticity is a major driver of treatment resistance, with therapies often pushing the glioblastoma ecosystem toward resistant states. She discussed variable responses to standard-of-care treatments and the co-evolution of tumor cells and their microenvironment at recurrence [23–24]. Preliminary, unpublished data from her group suggest that resistance mechanisms may be mediated by intrinsic or adaptive responses, warranting further validation in clinically relevant models [25–26]. Additionally, adaptive resistance may involve changes in the crosstalk between glioblastoma cells and surrounding non-neoplastic cells, particularly microglia [27]. While the talk emphasized that novel treatment strategies must address this inherent plasticity, allowing glioblastoma cells to transit into treatment-resistant states, outsmarting such ‘shapeshifting’ cancers will require truly innovative approaches.

***Linking CSC plasticity to therapeutic resistance - mechanisms and novel therapeutic strategies in hepatocellular carcinoma – Stephanie Ma*** from The University of Hong Kong, Hong Kong SAR, China, presented her research employing *in vivo* lineage tracing combined with single-cell RNA sequencing (scRNA-seq) to explore the heterogeneity and dynamic behavior of Prom1-positive (CD133-positive) HCC cells. Her findings indicate that Prom1 marks a subset of proliferative, tumor-propagating cells that possess CSC-like properties within HCC. Lineage tracing experiments demonstrated that these Prom1-positive cells undergo clonal expansion within primary tumors *in situ*. Functional assays revealed that labeled Prom1-positive cells exhibit enhanced tumorigenic capacity in 3D culture and allotransplantation models, with the potential to give rise to tumors of different lineages upon transplantation. Depletion of Prom1-positive cells significantly impeded tumor growth and reduced malignant characteristics across various HCC models. Further analysis through scRNA-seq uncovered heterogeneity within the Prom1-positive HCC population, revealing a trajectory toward dedifferentiation characterized by high proliferative and stemness traits. A conserved gene signature associated with the Prom1 lineage was found to predict poor prognosis in human HCC. Additionally, activated oxidative detoxification pathways appear to underpin the protective mechanisms that facilitate dedifferentiation and lineage propagation in these cells [28].

While Prom1/CD133 marks a critical subset of HCC cells exhibiting dedifferentiation and stemness traits, it is also expressed in regenerative tissues of the liver, intestines, and bone marrow. The widespread expression raises concerns regarding potential off-target effects and safety issues, which may limit the therapeutic potential of CD133-targeted strategies in clinical settings. Addressing tumor plasticity mediated by CD133 requires identifying molecular targets that are specifically expressed in CD133-positive HCC progenitor cells. Ma shared recent findings on the therapeutic potential of targeting SPINK1 [29], as well as repurposing existing drugs to inhibit MAP2, which has shown synergy with tyrosine kinase inhibitors in HCC treatment. Furthermore, her team identified AGPAT4 as an oncofetal protein that functions as a regulator of tumor lineage plasticity, metastasis, and resistance to sorafenib. Elevated activity of AGPAT4 promotes plasticity through increased conversion of lysophosphatidic acid (LPA) to phosphatidic acid (PA), leading to activation of downstream mTOR/S6K/S6 signaling pathways. Using an AAV8-mediated liver-directed approach in an immunocompetent HCC mouse model, inhibition of Agpat4 reduced tumorigenicity and stemness, while sensitizing tumors to sorafenib. Through a chemical biology approach, a cysteine-reactive compound targeting the Cys228 residue of AGPAT4 was identified, effectively inhibiting its acyltransferase activity. This compound demonstrated synergistic effects with sorafenib in suppressing HCC in patient-derived tumor xenograft models, including those resistant to sorafenib. Toxicological analyses indicated minimal side effects associated with this covalent inhibitor, opening promising new avenues for effective HCC therapy [30].

***Paracrine control of pancreatic cancer cell fates – Axel Behrens*** from Institute of Cancer Research, United Kingdom, described a complex network of communication between tumor cells that is central to cell fate decisions and progression of pancreatic ductal adenocarcinoma (PDAC). His laboratory previously demonstrated that the sustained suppression of BMP activity by the BMP antagonist Grem1, secreted by mesenchymal PDAC cells, is essential to maintain the fate of epithelial PDAC cells [31]. In a recent follow-up study, Behrens and colleagues identified Spp1, also known as Osteopontin, as a key regulator of mesenchymal cell fate in pancreatic cancer [32]. Spp1 was found to be expressed in epithelial PDAC cells, and its inactivation resulted in a phenotypic shift from mesenchymal to epithelial cell states. Consequently, inactivation of Spp1 led to a significant delay in tumorigenesis in murine PDAC models, and abolished metastasis formation. These findings reveal that the epithelial and mesenchymal cell fates within PDAC are governed by reciprocal paracrine regulation involving two soluble factors: Grem1 and Spp1.

***Decoding the mechanisms regulating cellular plasticity in breast cancer and aging – Shaheen Sikandar*** from University of California Santa Cruz, United States, presented recent findings from her laboratory investigating how pregnancy impacts cellular plasticity to decrease the risk of developing breast cancer. While several studies have explored the effects of aging or pregnancy in silo, the combined impact remains poorly understood. Sikandar’s team has established a comprehensive map of the long-term effects of pregnancy on the aging of stem/progenitor cells in the mammary gland. Their findings reveal that pregnancy counteracts age-induced imbalances in stem/progenitor cells, promoting a more normalized cellular landscape and inducing a differentiated cell state. Notably, they identified a rare population of IL-33-expressing hybrid cells characterized by high plasticity, which accumulate in aged nulliparous mice but are significantly diminished in aged parous mice. Functionally, their work also showed IL-33 regulates cellular plasticity and recapitulates aging phenotypes *in vitro* and *in vivo*. This collectively demonstrates that specific cellular subsets within normal and malignant tissues with high plasticity are responsible for distinct tumor-associated phenotypes [33].

## 4. Metabolic Reprogramming, Plasticity and Tumor Progression

***Exploiting metabolic vulnerability for therapeutic intervention in cancer – Wai Leong Tam*** from the Genome Institute of Singapore, A*STAR; and the Cancer Science Institute of Singapore, National University of Singapore discussed the concept of cancer as a metabolic disease, emphasizing that malignant transformation and progression involve a diverse array of metabolic reprogramming strategies [34]. He first explained how fatty acids are utilized in beta-oxidation following EMT, thereby promoting metastasis [35]. Tam then demonstrated that the essential amino acid methionine is critical for tumor initiation, primarily through its regulation of DNA and histone methylation [36]. Interestingly, emerging data suggest that tumor-initiating cells exhibit greater plasticity than previously thought; under certain nutrient-limiting conditions, they can harness ketone metabolism, highlighting this pathway as a potential therapeutic target for treating refractory or aggressive cancers. While much research has focused on the cell-intrinsic nutrient requirements of cancer cells, the influence of the tumor microenvironment and dietary factors has been underappreciated [37]. New findings from Tam’s lab reveal complex metabolic exchanges between cancer cells and surrounding cancer-associated fibroblasts (CAFs), which can be modulated through dietary interventions. Specifically, CAFs appear to regulate metabolite availability, such as aspartate, to induce niche-specific metabolic vulnerabilities, thereby opening additional avenues for the development of metabolic inhibitors to target tumor growth.

***Macrophage-mediated lipid transfer supporting treatment-resistant persister cells in glioblastoma – Loic Deleyrolle*** from Mayo Clinic, United States, highlighted that glioblastoma (GBM) remains one of the most intractable human cancers. Despite advances in genomics, single-cell technologies, and immunotherapies, nearly all patients experience relapse, driven by resistant tumor subpopulations that facilitate inevitable disease progression. Deleyrolle presented a compelling new perspective on how these resistant cells persist by co-opting metabolic support from the tumor microenvironment. His laboratory has long investigated the hierarchies and adaptive states underpinning GBM plasticity. Their latest work reveals that treatment-resistant persister cells (TRPCs) adopt a fundamentally distinct metabolic program compared to the bulk of therapy-sensitive tumor cells. While sensitive populations are predominantly glycolytic, TRPCs shift toward a lipid-dependent metabolic state, which sustains their survival and enables escape from standard treatments. Spatial multi-omics and functional analyses uncovered that TRPCs localize to specialized niches enriched with tumor-associated macrophages (TAMs). Rather than merely coexisting within an immunosuppressive microenvironment, TAMs are metabolically rewired to facilitate lipid supply, effectively supporting persister cells. This reciprocal partnership involves TRPCs attracting and supporting macrophages through chemotactic and pro-survival signals, such as the CCR2 and CSF1R pathways, while macrophages adapt to deliver lipid cargo. This immunometabolic cooperation establishes a protected niche that allows TRPCs to withstand therapy and drive tumor recurrence. Disrupting this metabolic homeostasis has shown profound tumor-inhibitory effects. Preclinical models demonstrated that interfering with lipid trafficking or macrophage recruitment remodels the tumor microenvironment, reduces persister cell survival, and delays disease progression. These findings identify a tractable therapeutic vulnerability within the TRPC-TAM axis. Patients with higher TRPC signatures exhibited significantly worse outcomes; however, intriguingly, individuals within this high-risk group who received statins, widely used lipid-lowering agents, showed improved survival, suggesting that statins may indirectly inhibit lipid trafficking within persister niches. Together, these insights reveal a novel metabolic dependency and present opportunities for drug repurposing and patient stratification. Beyond GBM, the broader implications are notable: treatment persistence is a hallmark of many cancers, and the principle uncovered here, that resistant cells co-opt immune cells to supply essential metabolites, may represent a general mechanism of therapy evasion. By reframing resistance and recurrence as a cooperative metabolic network, this work offers a significant conceptual advance with tangible therapeutic consequences. Building upon a body of research from Deleyrolle and colleagues examining tumor hierarchies, metabolism, and plasticity, the study leverages spatial multi-omics, preclinical models, and clinical data to move beyond descriptive observations towards a mechanistic and actionable framework. The identification of lipid-fueled persister niches supported by macrophage cooperation provides both a compelling biological insight and promising translational opportunities.

## 5. Cancer Cell Plasticity During Metastasis

***Plasticity of circulating tumor cell clusters in metastasis – Huiping Liu*** from Northwestern University, United States, discussed how cellular plasticity and stemness properties enable dynamic changes of circulating tumor cells (CTCs) during cancer dissemination, such as aggregation or cohesion of single CTCs into multicellular CTC clusters with 20–100 times higher metastatic propensity than the singles. Her research demonstrated that stemness glycoproteins on breast cancer stem cells, such as CD44 [38], CD81 [39], ICAM1 [40], and PlexinB2 [41], play a crucial role in driving CTC aggregation, particularly in metastasis of triple-negative breast cancer (TNBC). To investigate the glycosylation patterns in CTCs and their association with clinical outcomes, her team adopted multiple CTC analytical approaches, including CellSearch, Parsortix, flow cytometry, immunohistochemistry, and scRNA-seq using patient blood and tissue sections collected at the pre-treatment baseline and post-therapy time points. They discovered that chemotherapy-evasive CTC clusters are relatively quiescent, characterized by a specific loss of terminal sugar residues (α2,6-sialic acids) on glycoproteins. These CTCs exhibited dynamic hypo-sialylation in the bloodstream, with a marked reduction of the sialyltransferase enzyme ST6GAL1, which promotes cluster formation and cellular quiescence (proliferative dormancy), thereby enabling evasion of chemotherapy. Conversely, seeded tumor cells reacquire ST6GAL1 expression to facilitate metastatic colonization. Many adhesion proteins and stemness regulators, acting as substrates of ST6GAL1, drive CTC clustering and subsequent metastatic seeding [42]. Additionally, CTC clusters often associate with immune cells, particularly white blood cells (WBCs), which further support CTC stemness and immune suppression, thus promoting metastasis [43]. Importantly, neutralizing antibodies targeting these clustering drivers have been shown to prevent CTC cluster formation, enhance therapeutic responses, and eliminate lung metastasis in TNBC models.

***Molecular mechanisms of plasticity in intestinal regeneration and colorectal cancer – Karuna Ganesh*** from Memorial Sloan Kettering Cancer Center, United States, discussed her lab’s recent discoveries on cell state transitions and plasticity in colorectal cancer metastasis. During tumor progression, cancer cells dynamically reprogram their transcriptional output to enable microenvironmental adaptation, immune evasion, and therapy resistance. Understanding the molecular mechanisms that enable such emergent properties to arise during metastasis are essential for developing more effective therapies for advanced cancer; however, these mechanisms remain poorly understood. Using an unprecedented biospecimen trios comprising surgically resected patient normal colon tissue, primary colorectal tumors, and metastatic lesions profiled through scRNA-seq, along with matched patient-derived organoids, the Ganesh lab, in collaboration with the Dana Pe’er lab, recently discovered that while primary tumors are restricted to canonical intestinal stem cell-like states, metastases exhibit progressive cellular reprogramming into a highly conserved fetal gut progenitor state. This reprogramming enables lineage plasticity into non-canonical neuroendocrine and squamous states in metastasis and post-therapy, associated with aggressive behavior and poor clinical outcomes [44]. In their quest to identify mediators of this sequential phenotypic reprogramming, the team identified PROX1, a transcriptional corepressor whose expression peaks in the fetal state. PROX1 knockdown in primary tumor organoids with canonical gene expression induces the expression of fetal and non-canonical genes, suggesting that PROX1 functions to repress non-intestinal gene expression and safeguards lineage identity. Ganesh also discussed ongoing work aimed at uncovering mechanistic mediators of these cell state transitions, with the goal of developing therapies to block or reserve pro-metastatic plasticity.

## 6. Cancer Stemness and the Tumor Microenvironment

***Tumor initiating at the single cell level – lessons from normal development – Walid T. Khaled*** from the Cambridge Stem Cell Institute, United Kingdom, highlighted that despite significant advances in immune and targeted therapies, the molecular and cellular complexity of late-stage tumors remains a major obstacle to achieving a cure for cancer. This underscores the importance of focusing on the early stages of epithelial tumor initiation, identifying individuals at risk, and implementing prophylactic interventions to prevent tumor development. A key challenge in breast cancer prevention and treatment is our limited understanding of the dynamic cellular shifts within the breast and how these changes contribute to tumor initiation. To address this, Khaled’s team employed a combination of single-cell genomics, mouse models, and primary human samples to map cellular changes during homeostasis and tumor initiation [45–47]. Most recently, they completed a Human Breast Cell Atlas study using scRNA-seq to profile over 800,000 cells from 55 donors undergoing reduction mammoplasty or risk-reduction mastectomy. Notably, immune cells from BRCA1/2 mutation carriers exhibited a gene expression signature indicative of immune exhaustion, validated by immunohistochemistry, suggesting early immune escape mechanisms during tumor initiation. This atlas offers a valuable resource for developing strategies for early detection and prevention of breast cancer [48–49].

***Targeting tumor plasticity to combat immune evasion in liver cancer – Terence K. Lee*** from The Hong Kong Polytechnic University, Hong Kong SAR, China, previously reported on the roles of CD24 and CD47 as potential markers for liver cancer stem cells, functioning as “don’t eat me” signals, effectively suppressing immune responses in HCC. His team utilized a novel non-viral peptidic platform to create CAR-macrophages (pCAR-Ms) aimed at targeting cancer stemness through the induction of phagocytosis [50]. Using glypican-3 as a proof-of-concept target, they demonstrated that pCAR-Ms specifically and effectively induced phagocytosis *in vitro*. Remarkably, these engineered macrophages infiltrated tumors and suppressed HCC tumor growth in two xenograft models. In a c-MYC-overexpressing/p53 knockout mouse model of HCC, pCAR-Ms presented tumor antigens and reshaped the tumor microenvironment by activating T cells, thereby promoting an anti-tumor immune response.

Increasing evidence suggests that the activation of oncogenic pathways contributes to an unfavorable tumor immune microenvironment (TIME), leading to resistance to immunotherapy. To investigate this, Lee employed an antigen-expressing c-MYC-luciferase/p53 knockout HCC mouse model to identify key oncogenic pathways involved in immune evasion. Proteomic analyses revealed that wild-type KRAS signaling plays a critical role in this process by activating EGF-driven EGFR/MEK/ERK signaling. KRAS was found to inhibit intrinsic interferon-mediated MHC-I expression and impair extrinsic interferon-induced dendritic cell recruitment, resulting in diminished CD8+ T cell activity. These findings are clinically significant, as KRAS activation correlates with poor responses to PD-1 therapy. Further studies using animal models demonstrated that targeting the wild-type KRAS pathway in combination with PD-1 blockade increased intratumoral CD8+ T cell infiltration and improved survival [51].

## 7. Tumor Plasticity and Immune Evasion

***Sex hormones function to drive divergent glioblastoma trajectories through immune lineage-specific interactions – Justin D. Lathia*** from Cleveland Clinic, United States, presented his research on sex differences in glioblastoma in the context of immune interactions within the tumor microenvironment. It is well-documented that males are more frequently diagnosed with glioblastoma, and their tumors tend to be more aggressive [52]. His team previously identified key immune changes contributing to these differences, including increased myeloid-derived suppressor cells [53] and a higher prevalence of exhausted CD8+ T cells in the male tumor microenvironment [54]. However, the extent to which sex hormones drive this disparities remains unclear, and this has been an active area of cancer research in the context of immune regulation.

To further investigate the mechanisms underlying cellular plasticity in the tumor microenvironment, specifically the roles of additional cell types, sex hormones, sex chromosomes, and their interactions, his team employed preclinical mouse models. They examined the contribution of male sex hormones to glioblastoma growth through surgical (castration) and pharmacological (androgen receptor blockade with enzalutamide) methods of hormone neutralization. While previous studies in solid tumors suggested that testosterone neutralization reduced tumor growth [55], they found that in intracranial models, testosterone neutralization actually accelerated tumor progression [56]. Similar results were observed when non-brain tumors (such as melanoma and bladder cancer) were engrafted in the brain. Further, testosterone neutralization led to decreased activity of CD8+ T cells. Notably, mouse models lacking T cells did not exhibit increased tumor aggressiveness following testosterone suppression. They also observed that testosterone neutralization triggered an aberrant activation of the hypothalamus-pituitary-adrenal (HPA) axis, resulting in elevated glucocorticoid production. Treatment with glucocorticoid receptor antagonists mitigated the increased tumor aggressiveness caused by testosterone removal. Although no tumor cells were detected in the hypothalamus, unbiased profiling under testosterone-neutralizing conditions revealed increased signaling of interleukin-1 beta (IL-1β) and tumor necrosis factor (TNF). The aggressive phenotype observed with testosterone depletion was rescued in mice deficient for IL-1 receptor 1 (IL-1R1) and TNF receptors 1 and 2 (TNFR1/2).

Since glioblastoma typically develops later in life when testosterone levels decline, the team analyzed human tissue samples and observed a reduction in T cell infiltration within the male microenvironment, an effect not seen in females, validating their findings in mouse models. These results suggest a novel, brain-specific tumor-suppressive role for testosterone and highlight potential targets for next-generation immunotherapy strategies.

***Manipulating myeloid cell plasticity in glioma to enhance immunotherapy – Tyler E. Miller*** from Case Western Reserve University, United States, shared that gliomas are among the most treatment-resistant cancers, in large part due to an immunosuppressive microenvironment dominated by myeloid cells. He presented an integrated analysis combining scRNA-seq, chromatin accessibility, spatial transcriptomics, and patient-derived glioblastoma organoids to systematically define the phenotypic programs of these cells [57–58]. This work uncovered four core immunomodulatory programs: microglial inflammatory and scavenger immunosuppressive programs specific to brain tumors, and systemic inflammatory and complement immunosuppressive programs also present in non-central nervous system tumors. These states are shared across all myeloid cell types and are not determined by lineage or mutation, but are dynamically shaped by cues such as hypoxia, cytokines (IL-1β, TGF-β), and standard dexamethasone treatment. Importantly, the expression of these programs correlates with survival and response to immunotherapy, providing a framework to stratify patients and guide therapy. Mechanistic studies identified key transcriptional regulators and pathways, suggesting tractable points for intervention. Ongoing work in the Miller lab aims to directly manipulate these programs, such as reprogramming immunosuppressive phenotypes toward inflammatory states, minimizing dexamethasone-induced suppression, and developing preclinical platforms for targeted immunotherapies. This systematic approach provides both conceptual clarity and translational pathways to overcome myeloid-mediated resistance in glioma.

## 8. Novel Therapy in Stem Cells and Cancer

***Cellular heterogeneity and plasticity in normal prostate and prostate cancer – Dean Tang*** from Roswell Park Cancer Institute, United States, discussed the cellular heterogeneity and plasticity present in the normal prostate and prostate cancer (PCa), and their impact on tumorigenesis, progression and therapy resistance. The prostate is an epithelial glandular organ. In the normal human prostate, the basal cell compartment harbors primitive stem cells and intrinsically expresses neurogenic and mesenchymal gene expression profiles that can be linked to aggressive PCa subtypes [59]. The basal-cell layer also harbors unipotent basal progenitor cells. On the other hand, the prostate luminal layer is also heterogeneous containing both differentiated luminal cells and luminal progenitor cells, which they have shown to represent the preferred targets of tumorigenic transformation [60]. Their recent work has revealed that castration of the mouse prostate causses significant plasticity in luminal cells leading to a *de novo* castration-resistant epithelial population regulated by transcription factors AP1 and FOXQ1 [61].

In PCa, apparent cellular heterogeneity also exists in treatment-naïve tumors and stem cell-like PCa cells are phenotypically undifferentiated (*i.e*., AR^−/lo^PSA^−/lo^), relatively quiescent, and resistant to clinical therapies including castration and chemodrugs [62]. These PCa stem cells (PCSCs) preferentially express stem cell genes and epigenetic landmarks, can undergo asymmetric cell division and regenerate differentiated (PSA^+^) PCa cells, and become greatly enriched in treatment-failed tumors. PCSCs and PCa metastasis are positively regulated by BCL-2 [63] and NANOG [64] and negatively regulated by LRIG1 [65] and tumor-suppressive miRNAs miR-34a [66] and miR-141 [67]. Their work has prospectively pinpointed a population of PCa slow-cycling cells (PSCCs), which share many of the genes expressed in PCSCs and drive therapy resistance and tumor relapse *in vivo* through collective mesenchymal plasticity.

***Targeting cancer-selective metabolic vulnerabilities in MYC-amplified medulloblastoma – Sheila Singh*** from McMaster University, Canada, presented her research on MYC-driven medulloblastoma (MB), an aggressive pediatric brain tumor characterized by therapy resistance and disease recurrence. Her laboratory integrated data from unbiased genetic screening and metabolomic profiling to identify multiple cancer-selective metabolic vulnerabilities in MYC-driven MB tumor cells, which are amenable to therapeutic targeting. Among these targets, dihydroorotate dehydrogenase (DHODH), an enzyme that catalyzes *de novo* pyrimidine biosynthesis, emerged as a favorable candidate for therapeutic targeting. Mechanistically, DHODH inhibition acts on target, leading to uridine metabolite scarcity and hyperlipidemia, accompanied by reduced protein OGlcNAcylation and c-Myc degradation. Pyrimidine starvation evokes a metabolic stress response that leads to cell-cycle arrest and apoptosis. She further showed that an orally available small- molecule DHODH inhibitor demonstrates potent mono-therapeutic efficacy against patient- derived MB xenografts *in vivo*. The reprogramming of pyrimidine metabolism in MYC-driven medulloblastoma represents an unappreciated therapeutic strategy and a potential new class of treatments with stronger cancer selectivity and fewer neurotoxic sequelae [68]. Additionally, *de novo* pyrimidine synthesis has been found to be a targetable vulnerability in diffuse midline gliomas and IDH-mutant gliomas as well as MYC-amplified medulloblastoma, making a compelling argument that targeting DHODH and subsequent epigenetic remodeling could present a new strategy for treating certain undruggable brain cancers [69].
